# Prevention of Cytotoxic T Cell Escape Using a Heteroclitic Subdominant Viral T Cell Determinant

**DOI:** 10.1371/journal.ppat.1000186

**Published:** 2008-10-24

**Authors:** Noah S. Butler, Alex Theodossis, Andrew I. Webb, Roza Nastovska, Sri Harsha Ramarathinam, Michelle A. Dunstone, Jamie Rossjohn, Anthony W. Purcell, Stanley Perlman

**Affiliations:** 1 Department of Microbiology, University of Iowa, Iowa City, Iowa, United States of America; 2 Immunology Graduate Program, University of Iowa, Iowa City, Iowa, United States of America; 3 The Protein Crystallography Unit, Department of Biochemistry and Molecular Biology, School of Biomedical Sciences, Monash University, Clayton, Victoria, Australia; 4 Department of Biochemistry and Molecular Biology, Bio21 Molecular Science and Biotechnology Institute, University of Melbourne, Parkville, Victoria, Australia; University of California Irvine, United States of America

## Abstract

High affinity antigen-specific T cells play a critical role during protective immune responses. Epitope enhancement can elicit more potent T cell responses and can subsequently lead to a stronger memory pool; however, the molecular basis of such enhancement is unclear. We used the consensus peptide-binding motif for the Major Histocompatibility Complex molecule H-2K^b^ to design a heteroclitic version of the mouse hepatitis virus-specific subdominant S598 determinant. We demonstrate that a single amino acid substitution at a secondary anchor residue (Q to Y at position 3) increased the stability of the engineered determinant in complex with H-2K^b^. The structural basis for this enhanced stability was associated with local alterations in the pMHC conformation as a result of the Q to Y substitution. Recombinant viruses encoding this engineered determinant primed CTL responses that also reacted to the wildtype epitope with significantly higher functional avidity, and protected against selection of virus mutated at a second CTL determinant and consequent disease progression in persistently infected mice. Collectively, our findings provide a basis for the enhanced immunogenicity of an engineered determinant that will serve as a template for guiding the development of heteroclitic T cell determinants with applications in prevention of CTL escape in chronic viral infections as well as in tumor immunity.

## Author Summary

Enhancing the immune responses to pathogens is a chief goal of vaccine development. Here, we describe the development of an engineered CD8+ T cell epitope that elicits an immune response to the native epitope that is more potent than the one that occurs during the natural infection. We showed that this “improved” (heteroclitic) epitope protects against clinical disease and against cytotoxic T cell escape that frequently occurs in the immunodominant epitope expressed by the virus. We also performed structural analyses and showed that enhanced immunogenicity was associated with changes in the conformations of both the peptide and the region of the MHC class I molecule that is in close association with the peptide. These studies provide a model for designing T cell epitopes with enhanced immunogenicity that will be useful in vaccine development, with particular emphasis on diseases, such as HIV and hepatitis C, in which epitope mutation and escape is common.

## Introduction

Despite the antigenic complexity of microbes, primary pathogen-specific cytotoxic CD8+ T lymphocyte (CTL) responses are commonly directed to just one or a few determinants. Furthermore, even when multiple epitopes are targeted, distinct patterns of epitope hierarchy often emerge. Such immunodominant epitopes commonly elicit high-magnitude CTL responses characterized by potent cytolytic function, whereas subdominant determinants generate responses that are relatively lower in magnitude and often less efficacious. In general, potent anti-viral CTL strongly correlate with control of infection and less clinical disease. Viral progeny selected on the basis of CTL surveillance can evolve to evade T cell responses. This selective pressure results in mutations in immunodominant CTL determinants that abrogate recognition. CTL escape virus is commonly observed in humans and nonhuman primates infected with HIV-1, hepatitis C virus (HCV) or simian immunodeficiency virus (SIV) and its selection often correlates with disease progression [1]–[4]. Escape mutations may diminish binding to the restricting MHC class I molecule, interfere with T cell receptor (TcR) recognition or interfere with antigen processing [5]–[7]. Escape mutations are usually detected in epitopes targeted by CTL that exhibit high functional avidity because the corresponding potent CTL response exerts high selective pressure on the virus [2]. In some HIV-infected patients, CTL escape occurs without an associated enhancement of virus replication, suggesting that the mutations compromised virus fitness or, alternatively, the variant determinant elicited a *de novo* CTL response [8],[9]. Virus fitness is sometimes restored, with concomitant increased virus replication, when a second, compensatory mutation is selected [10],[11]. Collectively these results suggest that given the importance of virus diversification (CTL escape) in disease progression, suppression of selection or outgrowth of CTL escape variants should improve outcomes in persistently infected animals and humans.

Modulating the immunogenicity of subdominant CTL determinants could potentially lead to the development of more efficacious vaccines that are more broadly protective and prevent or minimize the appearance of variant viruses that have mutated in dominant epitopes targeted by high-avidity CTL responses. Enhancement strategies, which result in augmented responses to the native, subdominant epitope, have been described for both MHC class I and class II-restricted determinants, whereby the most common approaches involve generating a series of conserved and non-conserved mutations at MHC anchor residues, followed by an empiric determination of whether each individual substitution augments T cell effector function [12]. Furthermore, evaluating the effect of epitope enhancement *in vivo* has generally been achieved via heterologous infection systems [13]–[15]. Thus, while these results demonstrate proof of principle, direct evidence for enhanced protection against autologous microbial infection *in vivo* is lacking. The design of heteroclitic determinants in which non-MHC anchor residues are targeted for substitution are also usually determined empirically. Studies of heteroclitic tumor epitopes have demonstrated the clinical utility of such determinants [16],[17]. Notably, however, there are no well defined examples of viral epitopes which demonstrate enhanced immunity and the molecular basis for the enhanced immunogenicity is not well understood.

Potential interventions to directly manipulate host-pathogen interactions and thereby diminish CTL escape variant selection are often difficult to evaluate since most examples of CTL escape occur in infected humans or non-human primates. By contrast, mice persistently infected with mouse hepatitis virus (MHV) strain JHM (JHMV) serve as a useful system for investigating anti-viral CTL responses and CTL escape [18]–[20]. Two JHMV-derived CTL epitopes are recognized in C57BL/6 (B6) mice. The immunodominant H-2D^b^-restricted CTL epitope (S510, CSLWNGPHL, spanning residues 510–518 of the Spike (S) glycoprotein) elicits a high-magnitude, high-avidity CTL response that drives virus diversification during persistent infection [18]–[22]. A second subdominant CTL epitope, S598 (H-2K^b^-restricted, RCQIFANI, spanning residues 598–605 of the S glycoprotein) also elicits an appreciable CTL response [21]; however, S598-specific CTL exhibit ∼100-fold lower functional avidity and do not protect from CTL escape in S510. The presence of a readily mutable dominant and a subdominant epitope with high and low functional avidity, respectively, in JHMV-infected mice is useful for investigating both epitope enhancement and approaches to diminishing CTL escape. Consistent with this notion, we have previously shown that the introduction of a second dominant CD8 T cell epitope into the JHMV genome (GP33 from lymphocytic choriomeningitis virus (LCMV)) protected mice from the development of CTL escape in S510 and enhanced virus clearance [22].

Here we determined whether the CD8 T cell response to S598 could be enhanced so that it now elicited a more potent T cell response that protected mice against the development of CTL escape at S510 and subsequent clinical disease. We modified epitope S598 (S598_Q600Y_) such that it elicited a high-avidity CTL response and using the crystal structures of the H-2K^b^/S598 and H-2K^b^/S598_Q600Y_ complexes, determined the basis of this enhanced immune response. We then introduced this more immunogenic S598 epitope into a recombinant version of JHMV and showed that these high-avidity S598-specific CTLs protected against escape variants in the immunodominant S510 epitope. Immunization with the modified peptide resulted in an improved response to the native S598 epitope, demonstrating a true heteroclitic effect and suggesting that this strategy may have clinical applications for reducing viral titer and preventing CTL escape during chronic infections.

## Results

### Induction of protective T cell responses with a heteroclitic virus

As we have previously observed with other MHC/Cys-containing peptide complexes [19],[23], complexes did not readily form unless the cysteine of the S598 peptide (RCQIFANI) was modified with L-α-aminobutyric acid (Aba, an isostereomer of cysteine). The Aba-modified peptides maintained immunogenicity since a higher frequency of splenic CD8+ T cells from JHMV-immune mice reacted to Aba-modified S598 peptide, relative to unmodified S598 peptide (**Figure S1A**).

**Figure 1:**
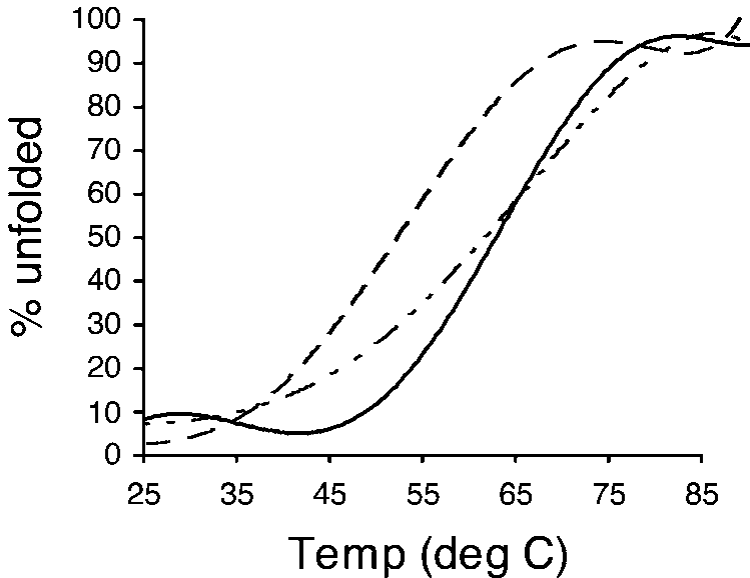
Thermal stability of the S598 and S598_Q600Y_ epitopes in complex with H-2K^b^. Circular dichroism (CD) analysis of S598-Aba (dashed line), S510-Aba (variable dash line) and S598_Q600Y_-Aba (solid line) complexed with H-2K^b^. CD spectra were measured as described in Materials and Methods.

**Figure 2:**
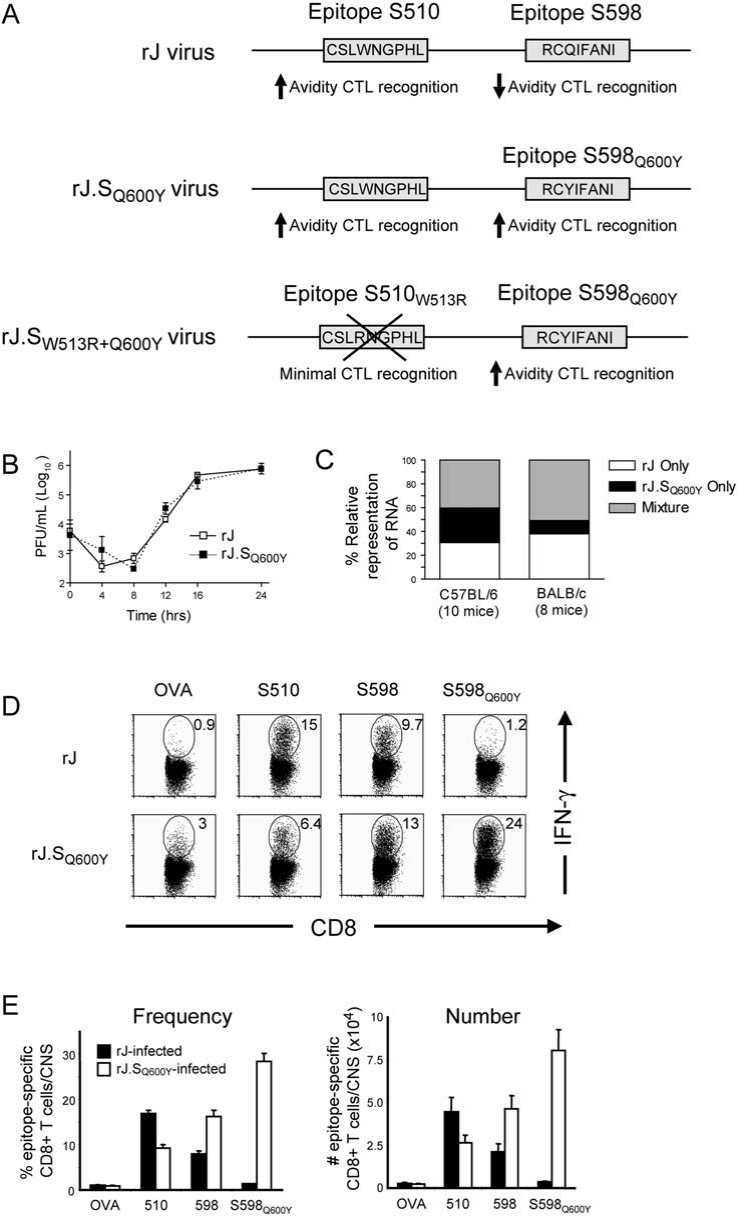
Fitness of and CTL responses elicited by rJ and rJ.S_Q600Y_. (A) Schematic depictions of recombinant wild type JHMV (rJ), recombinant JHMV encoding the Q600Y substitution (rJ.S_Q600Y_) and recombinant JHMV encoding the S598_Q600Y_ substitution in the context of an S510 CTL escape mutation (S510_W513R+Q600Y_). (B) Equivalent *in vitro* growth kinetics among rJ and rJ.S_Q600Y_ viruses. 17Cl-1 cells were infected with rJ or rJ.S_Q600Y_ at a multiplicity of infection (MOI) of 1.0. Cells and supernatants were harvested at the indicated times and titers were measured by plaque assay as described in Materials and Methods. Data are representative of two independent experiments. (C) Equivalent *in vivo* fitness among rJ and rJ.S_Q600Y_ viruses. Five week old B6 and BALB/c mice were infected with virus mixtures consisting of equal PFU of rJ and rJ.S_Q600Y_ variant virus. Seven days p.i., total RNA was harvested from the brains of mice and relative representation of virus template was determined via RT-PCR and direct sequencing of PCR products. The relative proportion of animals in which only rJ, only rJ.S_Q600Y_, or a mixture of the two viruses is shown. (D) High-magnitude, unidirectional cross-reactivity. Representative dot plots demonstrating the frequency of epitope-specific CD8 T cells in a mouse infected with rJ (top panels) or rJ.S_Q600Y_ (bottom panels). Numbers represent the frequency of epitope-specific CD8 T cells among total CD8 T cells recovered from the brains of mice 7 days p.i. (E) Summaries of the frequency (left panel) and absolute number (right panel) of epitope-specific CD8 T cells recovered from the brains of rJ and rJ.S_Q600Y_-infected mice 7 days p.i. Data shown in D represent mean±SEM for 4 independent experiments.

Next, we used the consensus H-2K^b^ binding motif [24] to engineer a novel, high-avidity S598 CTL epitope. Importantly, Gln-3 diverges from the consensus H-2K^b^-restricted ligand binding motif, in which a tyrosine is often present at position 3 [24],[25]. Therefore, we substituted a glutamine residue for tyrosine (Q600Y, CAA to TAT, RCYIFANI) at position 3 of the determinant with the aim of creating a peptide that bound more tightly to H-2K^b^. Stability of the H-2K^b^/S598 and H-2K^b^/S598_Q600Y_ complexes was assessed by circular dichroism (CD). As shown in **Figure 1**, H-2K^b^/S598_Q600Y_ was considerably more thermostable than the native complex (Tm 54°C vs 64°C). To probe the biological properties of the Q600Y substitution, we used reverse genetics to engineer a recombinant version of JHMV expressing the S598_Q600Y_ epitope (**Figure 2A**). Recombinant viruses encoding this substitution replicated as efficiently as wild type JHMV (rJ) *in vitro* during one-step growth kinetics analyses and *in vivo* virus competition assays (**Figure 2B, C**). The immunogenicity of S598_Q600Y_ was assessed by intracellular expression of IFN-γ by central nervous system (CNS)-derived lymphocytes. Since Cys-containing peptides are often diminished in ability to elicit a CTL response, we included a reducing agent (TCEP, Tris[2-carboxyethyl] phosphine) in the cultures. TCEP enhanced the stimulatory capacity of the S598 peptides (**Figure S1B**), indicating that a proportion of the unmodified S598 peptide stock had undergone oxidation. Thus, we stimulated CNS- and spleen-derived CTL *ex vivo* in the presence of 500 µM TCEP, a concentration consistent with other work with Cys containing peptides [23],[26].

The S598_Q600Y_ epitope elicited a CTL response in the CNS of rJ.S_Q600Y_ infected mice, with nearly 30% of all CD8 T cells recognizing the determinant (**Figure 2D**). In addition, CTL primed by S598_Q600Y_ cross-reacted with the native S598 determinant. The converse was not true, however, as cells primed by the native epitope failed to produce IFN-γ when stimulated with S598_Q600Y_ peptide (**Figure 2D**). Of note, the CTL response to the dominant D^b^-restricted S510 epitope in rJ.S_Q600Y_-infected mice was diminished relative to responses in mice infected with wildtype rJ virus (**Figure 2D, E**).

**Figure 3:**
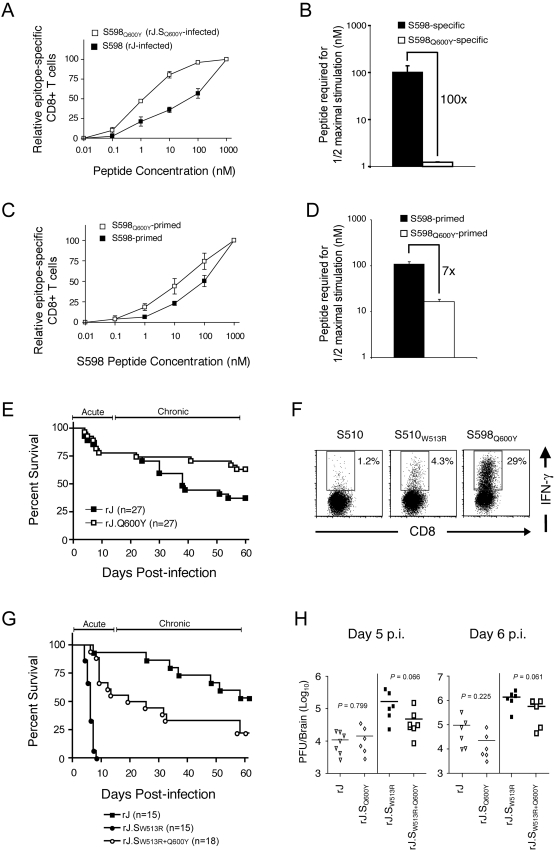
S598_Q600Y_ elicits a CTL response with higher functional avidity than wild type S598, protects from clinical disease in the context of an S510 CTL escape mutation and enhances virus clearance. (A) Functional avidity analysis of CD8 T cell populations primed by native S598 determinant or modified S598_Q600Y_ determinant. Five week old B6 mice were infected with rJ or rJ.S_Q600Y_ variant virus. Seven days p.i., CNS-derived mononuclear cells were harvested and cell aliquots were stimulated *ex vivo* with 10-fold serial dilutions of the native S598 peptide (for rJ-infected) or S598_Q600Y_ peptide (for rJ.S_Q600Y_-infected). Data are normalized to the frequency of determinant-specific cells detected using 1 µM of peptide. (B) Concentration of peptide required to elicit a half-maximal response during *ex vivo* cell stimulation. (C) Functional avidity of cross-reactive S598_Q600Y_-primed CTL. Five week old B6 mice were infected with rJ or rJ.S_Q600Y_, CNS-derived mononuclear cells were harvested and cell aliquots were stimulated *ex vivo* in the presence of 500 µM TCEP with 10-fold serial dilutions of only the native S598 peptide. Data are normalized to the frequency of determinant-specific cells detected using 1 µM of peptide. (D) Concentration of peptide required to elicit a half-maximal response during *ex vivo* cell stimulation. Data for A–D represent mean±SEM for 4 independent experiments. (E) Survival analysis of maternal antibody-protected suckling mice infected with rJ or rJ.S_Q600Y_ variant viruses. Suckling mice were infected at 10 days of age and nursed on dams that had been previously immunized with virulent JHMV. To control for the delivery of protective maternal antibody, one-half of each litter was infected with rJ while the other half was infected with rJ.S_Q600Y_. Data represent results from 10 litters derived from multiple independent JHMV-immune dams. Numbers in parentheses indicate the number of pups infected with each virus. Mice surviving the acute infection (day 0–14 p.i.) were monitored to day 60 p.i. and scored as deceased following development of hind limb paralysis/paresis. (F) Frequency of epitope-specific CD8 T cells in the brain of an rJ.S_W513R+Q600Y_-infected mouse. Seven days p.i., CNS-derived mononuclear cells were harvested, stimulated *ex vivo* with the indicated peptides and stained for CD8 and intracellular IFN-γ as described in Materials and Methods. The frequency of cells that produced IFN-γ in response to an irrelevant peptide (OVA (chicken ovalbumen, not shown) was equivalent to the S510-specific response. (G) Survival analysis of suckling mice infected with rJ, rJ.S_W513R_ or rJ.S_W513R+Q600Y_. One-third of the pups in an individual litter were infected with each virus. Data represent results from 6 litters derived from multiple independent JHMV-immune dams. Numbers in parentheses indicate the number of pups infected with each virus. Mice surviving the acute infection (day 0–14 p.i.) were monitored to day 60 p.i. and scored as deceased following development of hind limb paralysis/paresis. (H) Virus titers in the brains of maternal antibody-protected suckling mice 5 or 6 days following infection with rJ, rJ.S_Q600Y_, rJ.S_W513R_ or rJ.S_W513R+Q600Y_ viruses. One-fourth of the pups in an individual litter were infected with each virus. At the indicated day p.i., brains were aseptically harvested, homogenized in sterile PBS and clarified by centrifugation. Supernatants were collected and infectious virus was titered on Hela-MHVR (Hela cells transfected with CEACAM1, the JHMV receptor [59]) as previously described [27]. Symbols on graph represent individual mice assayed from multiple independent litters. The limit of detection (LOD) for the assay is 80 PFU/brain. Nonpaired, two-sided Student's *t* tests were used for statistical analyses.

Next, we assessed the relative functional avidity of CTL populations primed by native S598 and S598_Q600Y_ determinants, as a surrogate measure of the potency of the anti-virus CTL response *in vivo*. CNS-derived mononuclear cells were harvested from mice infected with rJ or rJ.S_Q600Y_ and examined for IFN-γ expression after stimulation *ex vivo* in the presence of 10-fold dilutions of the appropriate peptide. Cells primed to the native S598 epitope (cells harvested from rJ-infected mice) required approximately 100-fold more peptide than did S598_Q600Y_-primed cells to elicit a half maximal response (100 nM vs. 1 nM, **Figure 3A,B**).

Because we observed that a subpopulation of S598_Q600Y_-primed cells cross-reacted with the native determinant, we next determined whether this subpopulation also exhibited high functional avidity. For this purpose, we isolated cells from the CNS of rJ- and rJ.S_Q600Y_-infected adult B6 mice and stimulated them with 10-fold dilutions of native S598 peptide. S598_Q600Y_-primed cross-reacting cells exhibited 7-fold higher functional avidity, relative to those primed by the native determinant (**Figure 3C, D**) and are therefore distinct from CTL primed by the native S598 determinant. Consistent with the presence of a distinct population of cells, there were modest differences in Vβ chain utilization when total populations from rJ and rJ.S_Q600Y_-infected mice and when total and cross-reacting cell populations from rJ.S_Q600Y_-infected mice (**Figure S2**) were compared. In all instances, Vβ5.1/5.2 expression was relatively over-represented, but some Vβ elements were preferentially utilized by specific responding populations (Vβ11, Vβ13 and Vβ14). Also consistent with this observation, alanine scanning mutagenesis of the two determinants revealed that the CTL response to each cognate peptide following infection was also subtly different reflecting the altered repertoire. The CTL response to each cognate peptide was very sensitive to mutation at every position except 2 and 9 although mutations in the S598_Q600Y_ determinant were tolerated slightly better than changes in the S598 epitope (**Figure S2C–E**).

If CTL recognizing S598_Q600Y_ exhibit high functional avidity *in vivo*, they should protect from CTL escape in S510 and might select for S598_Q600Y_ CTL escape variants. Diversification at S510 is observed in pups infected with neurovirulent JHMV at 10 days of age and nursed by JHMV-immune dams [27]. These mice are largely protected from developing acute lethal encephalitis, but a variable percentage (30–90%) later develop a demyelinating encephalomyelitis. Infectious virus isolated from these mice with late onset clinical disease is mutated in S510, resulting in enhanced virus replication [20], with demyelination occurring during the process of virus clearance [28]. Thus, we next infected maternal antibody-protected suckling mice with rJ or rJ.S_Q600Y_ viruses and monitored persistently infected mice for the development of clinical signs for 60 days post infection (p.i.). The presence of the highly immunogenic S598_Q600Y_ epitope did not protect mice from acute encephalitis (**Figure 3E**), perhaps because the presence of the improved S598 epitope was accompanied by a diminished response to S510 (**Figure 2D**). However, we found that among survivors (defined as survival past day 14 p.i.) there was a significant reduction in the incidence of clinical disease as well as in the development of CTL escape in S510 (**Table 1**). Additionally, S598_Q600Y_ did not undergo CTL escape in mice persistently infected with rJ.S_Q600Y_, even though single nucleotide changes in the region of the S glycoprotein gene encoding the S598 determinant could potentially result in fifty-one CTL escape mutations. Thus, as expected, the “improved” S598_Q600Y_ epitope was protective *in vivo* in infected mice, likely because S598_Q600Y_-specific CTL are present in higher numbers and exhibit higher functional avidity than the native S598-specific response.

**Table 1:** Incidence of clinical disease and development of CTL escape in rJ and rJ.S_Q600Y_ infected mice.

Index Virus (survivors)^a^	% Clinical Disease^b^	S510 Escape Symptomatic^c^	S510 Escape Asymptomatic^c^	S598 Escape: (symptomatic and asymptomatic)
rJ (n = 19)	58 (11/19)	10/11	8/8	0/19
rJ.S_Q600Y_ (n = 19)	21 (4/19)^a^	0/4	0/15	0/19

a Numbers that survived acute infection (days 0–14 p.i.).

b χ^2^ = 5.39, *P*<0.025 for rJ versus rJ.S_Q600Y_.

c χ^2^ = 32, *P*<0.0001 for rJ versus rJ.S_Q600Y_.

### S598_Q600Y_-specific CTL are protective in mice infected with S510 CTL escape virus

Since this lack of mutation at S598_Q600Y_ might reflect enhanced suppression of virus replication mediated by co-dominant CTL responses directed against S510 and S598_Q600Y_, we developed a recombinant virus encoding S598_Q600Y_ in the context of a common S510 CTL escape mutation, S510_W513R_ (**rJ.S_W513R+Q600Y_,** **Figure 2A**). The CTL response is predicted to be largely directed at S598_Q600Y_ in mice infected with this virus. The W513R mutation (position 4 substitution in S510 epitope, CSLRNGPHL) occurs in 13% of all CTL escape variants [19],[20],[22],[29],[30], and completely abrogates native S510 CTL recognition [31]. We verified that virus-specific CTL responses were focused on S598_Q600Y_ in adult B6 mice infected with rJ.S_W513R+Q600Y_ (**Figure 3F**). To examine the phenotype of the S598_Q600Y_/S510_W513R_ double mutant, we infected antibody-protected suckling B6 mice with this virus and appropriate controls and monitored mice for survival (**Figure 3G**). As expected, 93.3% of mice infected with rJ survived the acute infection (day 0–14 p.i.). All mice infected with rJ.S_W513R_ developed fatal encephalitis but, in marked contrast, 66.6% of mice infected with rJ.S_W513R+Q600Y_ survived. We also found that survival correlated with virus clearance (**Figure 3H**). Relative to rJ-infected mice, replication was suppressed in mice infected with virus encoding the S598_Q600Y_ epitope and greatly elevated in mice infected with rJ.S_W513R_. In mice infected with rJ.S_W513R+Q600Y_, virus titers were intermediate between rJ and rJ.S_W513R_. Thus, the presence of the heteroclitic S598_Q600Y_ determinant contributed to suppression of virus replication and to increased survival, even when the high-magnitude, high-avidity CTL response to S510 was largely abrogated.

Surprisingly, S598_Q600Y_ still did not undergo sequence diversification in mice that survived the rJ.S_W513R+Q600Y_ infection (**Table 2**). This result was unexpected, as the majority of CTL in the rJ.S_W513R+Q600Y_-infected CNS specifically target the S598_Q600Y_ determinant and exhibit high functional avidity (**Figure 3A–C**). One possible explanation for this result is that S598 is not as plastic as S510, even though both determinants are derived from a region of the spike gene that is hypervariable and even deleted in some strains of MHV [32].

**Table 2:** Incidence of clinical disease and CTL escape in rJ and rJ.S_W513R+Q600Y_ infected mice.

Index Virus (survivors)^a^	% Clinical Disease^b^	S510 Escape Symptomatic^c^	S510 Escape Asymptomatic^c^	S598 Escape: (symptomatic and asymptomatic)
rJ (n = 14)	43 (6/14)	5/6	3/8	0/14
rJ.S_W513R+Q600Y_ (n = 10)	60 (6/10)	0/6	0/4	0/10

a Numbers that survived acute infection (days 0–14 p.i.).

b χ^2^ = 1.50, *P* = 0.221 for rJ versus rJ.S_W513R+Q600Y_.

c χ^2^ = 8.57. *P* = 0.0034 for epitope S510 CTL escape in rJ versus rJ.S_W513R+Q600Y_.

### Enhanced recognition of S598 by cells primed to S598_Q600Y_

**Figure 4:**
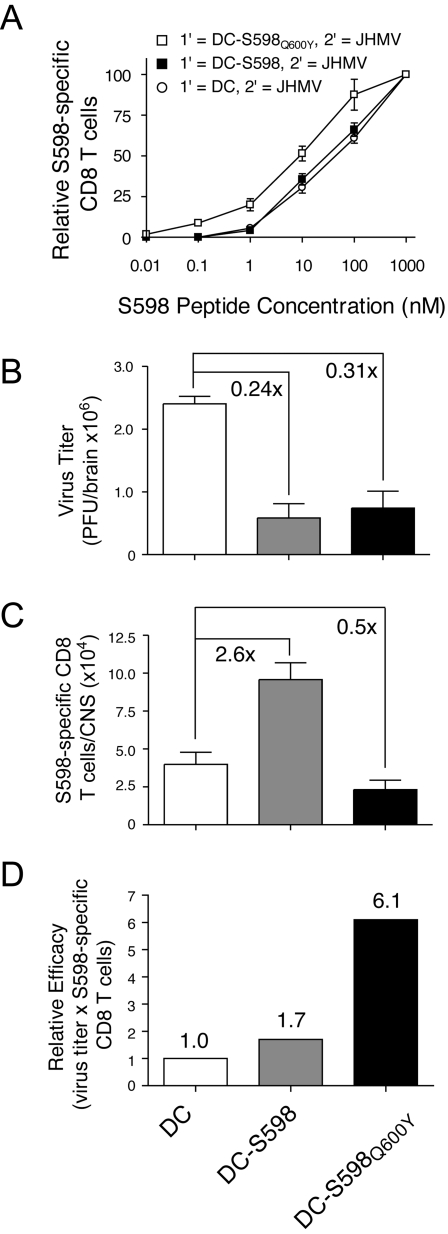
Functional avidity and protective capacity of cross-reactive, S598_Q600Y_-primed CTL. (A) Functional avidity analyses of S598- and S598_Q600Y_-primed CTL. Groups of 4-week-old B6 mice were vaccinated with LPS-matured bone marrow-derived DC pulsed with peptides corresponding to S598_Q600Y_, native S598 or no peptide. Seven days following DC vaccination, mice were intranasally infected with 8×10^4^ PFU of non-recombinant JHMV. On day 7 p.i., CNS-derived mononuclear cells were stimulated *ex vivo* with 10-fold serial dilutions of native S598 peptide. Data are normalized to the frequency of S598-specific cells detected using 1 µM of peptide. Data represent mean±SEM for 3 independent experiments. (B–D) Virus titers and magnitude of CTL response. Brains were aseptically removed from individual DC-vaccinated mice 7 days following JHMV challenge. One-half of the brain was homogenized and clarified for viral titer determination (B) while the other half was used to determine the numbers of S598-specific CTL (C). The relative efficacy of S598-specific CTL (D) was derived by calculating the product of virus titers and absolute numbers of S598-specific CTL for each individual mouse. Data in B–D represent results from six individual mice analyzed in two independent experiments.

To determine whether cells primed to S598_Q600Y_ that cross-react with the native S598 determinant were more protective *in vivo* than S598-primed cells, we vaccinated mice with bone marrow-derived dendritic cells (BMDC) alone, or BMDC pulsed with peptides corresponding to S598 or S598_Q600Y_ (**Figure 4**). Seven days later, mice were challenged via intranasal inoculation of 4×10^4^ PFU of wild type, non-recombinant JHMV. Similar to results observed following rJ.S_Q600Y_ infection (**Figure 3**), CTL primed via DC-S598_Q600Y_ vaccination exhibited higher functional avidity when reacted against the native S598 determinant when compared to those arising after DC-S598 vaccination **(** **Figure 4A** **)**. In other experiments, we examined the survival of mice vaccinated with each determinant, but we observed no significant differences between groups (data not shown), probably because mortality is largely CD4 T cell-mediated in adult mice with acute encephalitis [33],[34]. In terms of virus titers, vaccination with either the native or enhanced S598 determinants resulted in ∼70–80% reduction in virus burden compared to mice that received un-pulsed BMDC (**Figure 4B**). When we examined the frequency and numbers of virus-specific CTL in the CNS of these same mice, we detected markedly fewer S598-specific CTL in the CNS of mice that received BMDC pulsed with S598_Q600Y_ peptides (**Figure 4C**). Calculation of the product of virus titers and CTL numbers within individual mice, as an approximate measure of CTL potency, indicated that the S598-specific CTL in S598_Q600Y_-coated BMDC vaccinees were ∼6-7-fold more efficacious on a per cell basis (**Figure 4D**). Thus, S598-specific CTL induced by the S598_Q600Y_ determinant show similar enhancement in function compared to S598-primed cells, whether measured *in vitro* (**Figure 3C,D**) or *in vivo* (**Figure 4D**).

### Structures of the wild-type and engineered S598 H2K^b^ complexes

**Figure 5:**
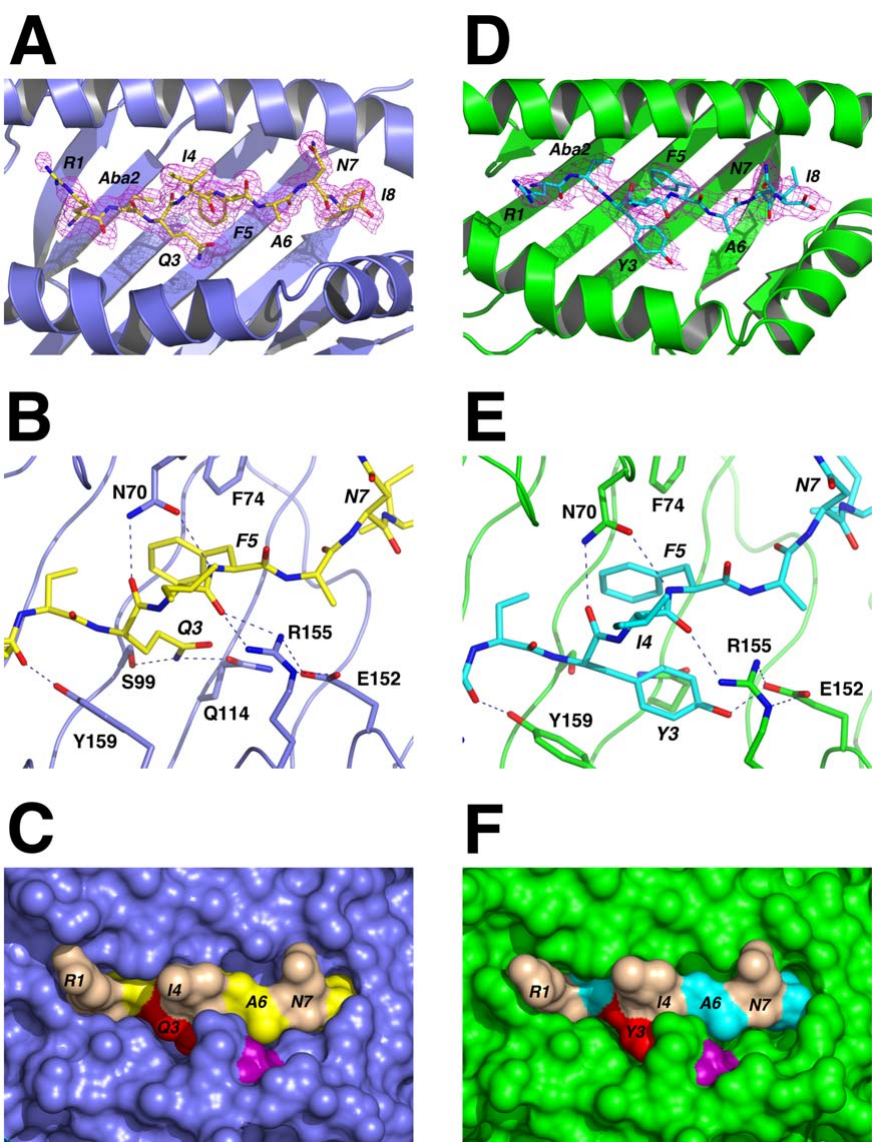
Refined structures of WT and Q600Y S598-Aba bound to H-2K^b^. (A) View of the H-2K^b^ antigen binding cleft from above. The HC is shown as a cartoon representation and coloured slate. The peptide is in stick format with carbon atoms coloured yellow. The final 2*F_o_-F_c_* map density for the peptide contoured at 1.0 σ is shown as a magenta mesh. (B) Detail of the antigen binding cleft displaying key interactions (dashed lines) between H-2K^b^ and S598-Aba in the region surrounding position 3 of the peptide. Selected residues of the HC are drawn in stick format (slate carbon atoms). Peptide residues are labelled in italics. (C) Surface representation of the H-2K^b^/S598-Aba complex as seen from above. Peptide residues Arg-1, Ile-4 and Asn-7 are coloured wheat. Position 3 of the peptide (Gln) is coloured red and the HC residue E-152 is purple. *D*, *E* and *F*, Equivalencies to A, B and C, respectively, for the H-2K^b^/S598_Q600Y_-Aba structure. In these panels the HC is drawn in green and the peptide in cyan.

While these studies clearly demonstrated that S598_Q600Y_ is heteroclitic, they did not provide a mechanism for the immune enhancement. To address this, we determined the crystal structures of the H-2K^b^/S598 (PDBid 2ZSV, Protein Data Bank Japan (http://www.pdbj.org/)) and H-2K^b^/S598_Q600Y_ (PDBid 2ZSW) complexes to 1.8 Å and 2.8 Å resolution respectively. The structure of H-2K^b^/S598 consists of two heterodimers in the asymmetric unit (r.m.s.d. of 0.18 Å for Cα atoms), with the S598 peptide clearly bound in the antigen binding cleft of the heavy chains (HC, **Figure S3A**). The two peptide copies display a virtually identical configuration with root mean square deviation (rmsd) values of only 0.09 Å for all peptide atoms (0.05 Å for Cα atoms). The mode of S598 and S598_Q600Y_ binding within the Ag-binding cleft is unambiguous, with the exception of Arg-1, whose side chain is partially disordered (**Figure 5A,D** **; Figure S3C**). The S598 peptide adopts an extended conformation, with the side chains of Arg-1, Ile-4 and Asn-7 extending prominently out of the cleft (**Figure 5C**). Ala-6 is also largely solvent exposed with its side chain pointing towards the α2 helix, while the side chains of Cys (Aba)-2, Gln-3, Phe-5 and Ile-8 are buried within the cleft. While the cysteine analogue's side chain is not involved in any hydrophilic interactions, there are a number of suitably positioned hydrogen bonding partners (Glu-24, Tyr-45 and Asn-70), with which the original thiol side chain could potentially interact (**Figure S3B; Table S2**).

In addition to main chain interactions across the length of the peptide, S598 is anchored to the MHC via the side chains of Gln-3, Phe-5 and Ile-8. Gln-3 forms hydrogen bonds with the Ser-99 and Gln-114 of the HC **(** **Figure 5B** **)**, while Phe-5 and Ile-8 are buried within hydrophobic pockets. In addition, the side chains of Phe-5 and Gln-3 pack against each other and between the aromatic rings of HC residues Tyr-159 and Phe-74, constraining the peptide's backbone conformation at those positions.

The structure of H-2K^b^/S598_Q600Y_ consists of four heterodimers in the asymmetric unit (rmsd of 0.09 Å for heavy chain (HC) Cα's), the four copies of the peptide adopting virtually identical conformations (rmsd of 0.12 Å for all peptide atoms and 0.05 Å for Cα atoms). S598_Q600Y_ displays the same conformation as the S598 determinant (the average rmsd for peptide Cα atoms between the two structures is 0.24 Å) and forms equivalent interactions with the MHC. The only prominent structural difference is observed at the mutated position (Q3→Y3) (**Figure 5B,E** **,** **6A,B**). In contrast to Gln-3, the Tyr-3 side chain is oriented towards the α-2 helix rather than the floor of the cleft. Consequently, the interactions with Ser-99 and Gln-114 of the HC are lost and instead the tyrosine's hydroxyl group forms a hydrogen bond with the side chain of Glu-152 (**Figure 5E**). The side chain of Tyr-3 also forms a number of close contacts with the residues in its immediate environment, specifically HC residues Gln-114, Arg-155, Leu-156 and Tyr-159, as well as intramolecular interactions with Phe-5 (**Table S2**). In addition to Tyr-3, deviations of potential significance (>0.5 Å) in the peptide structures that are attributable to the Q660Y mutation are observed at Ile-4 and Phe-5.

**Figure 6:**
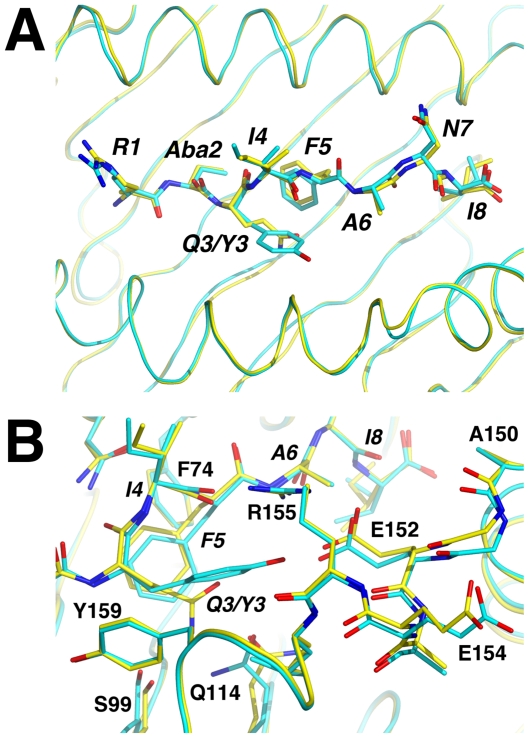
Comparison of the WT and Q600Y S598-Aba complexes with H-2K^b^. Superpositions were carried out using the Cα atoms of HC residues 1–176. (A) View of the H-2K^b^ antigen binding cleft from above. The HC backbone is shown in ribbon format and the peptide residues as sticks. The index determinant is drawn with yellow carbons and the Q600Y variant with cyan. This colour scheme is extended to include the HC carbon atoms of the same structure. (B) Detail of the antigen binding cleft showing conformational differences between the WT and Q600Y structures in the region surrounding position 3 of the peptide.

Overall the S598 determinant displays greater complementarity for the antigen binding cleft of H-2K^b^ in its N-terminal region, with few stabilizing interactions observed between the HC and positions 6 and 7. (**Figure S3B, D; Table S2**). Nevertheless, a pocket is observed between the α-2 helix and the peptide near position 3 in the index structure that is filled by the steric bulk of Tyr-3 in the Q600Y structure **(** **Figure 5C,F** **)**. This increase in surface complementarity and the greater number of observed interactions resulting from the Q3→Y3 mutation would account for the enhanced thermostability (∼10°C) measured for the H-2K^b^/S598_Q600Y_–Aba complex by circular dichroism (**Figure 1**). This increase in complementarity of the MHC for S598_Q600Y_ and the greater stability of the resulting complex were predicted from comparisons of the WT determinant complex with existing structures of H-2K^b^ bound with peptides similar to S598 and possessing the consensus tyrosine anchor residue at position 3, as well as another aromatic residue at position 5.

With respect to the HC, the α-1 and α-2 domains of the two structures superimpose well with an rmsd of 0.37 Å for Cα atoms (residues 1–176). Nevertheless, significant deviations (>0.5 Å) are observed between the two structures at a number of positions in the region of the antigen binding cleft. In particular, changes in the conformation of Ser-99, Gln-114, and Gly-151-Glu-154 are associated with the bound peptides. Changes in the side chain conformations of Ser-99 and Gln-114 in the Q600Y complex structure are consistent with the loss of hydrogen bonding interactions with position 3. In the wild type peptide structure, Glu-152 forms a salt bridge with Arg-155, the guanadinium group of which also forms a hydrogen bond with the main chain carbonyl group of the peptide's Ile-4. While these interactions are maintained in the Q600Y complex, the side chain of Glu-152 displays a conformational shift consistent with the formation of a hydrogen bond with the peptide's Tyr-3 (**Figure 6**). Interestingly the region of the α-2 helix around Glu-152 (Gly-151 - Glu-154) also displays significant main chain and side chain deviations between the two structures (rmsd of 0.77 Å for Cα atoms; **Figure 6**). Thus, consistent with the functional analyses, the structure of the heteroclitic variant of the S598 epitope displays relatively small changes in conformation yet the combination of these subtle changes in the TcR accessible residues and the structural landscape of the MHCp in addition to the enhanced stability of the complex lead to more efficacious CTL responses.

## Discussion

We describe the identification of a heteroclitic determinant that enhances recognition by virus-specific CD8 T cells, and use the crystal structure of the new determinant (S598_Q600Y_) to provide a basis why it elicits an enhanced CTL response. Comparison of S598 to the consensus binding motif suggested a suboptimal interaction with the H-2K^b^ molecule at the secondary anchor position (Gln-3) and, consequently, an approach to enhance the immunogenicity of the determinant. Replacement of the Gln-3 with Tyr-3 (Q600Y) did, indeed, result in an determinant with increased thermostability without diminishing the CD8 T cell response. Most strikingly, the Q600Y change resulted in subtle changes in the conformation of the α-2 helix locally in the vicinity of Glu-152. These subtle changes are likely critical for the enhanced TcR recognition that we detected.

S598_Q600Y_ elicited a response with higher functional avidity to both the cognate and native determinants than S598, and this was not reflected in differential Vβ usage. The T cell response to S598 in rJ-infected mice is very diverse [35]. As assessed by Vβ usage, the response to S598 and S598_Q600Y_ in rJ.S598_Q600Y_ was similarly diverse with only modest differences noted when cells from mice primed by S598 and S598_Q600Y_ were compared. While we cannot exclude the possibility that cross-reacting S598-specific cells primed by S598_Q600Y_ are biased for Vβ chains not analyzed in this study, it is more likely that the fine specificity of the complementarity-determining region 3 (CDR3) determines their greater affinity for H-2K^b^/S598. Although an increase in stability of the MHC class I/peptide complex is not generally expected to enhance TcR affinity for the complex, similar results have been observed in mice immunized with analogues to a common tumor antigen [36].

One unexpected result was that S598 exhibited both low MHC class I and low TcR avidity. Previous studies showed that this determinant exhibited low functional avidity [21], but it was not known whether this reflected low binding to the MHC class I or to the TcR. Assuming that low affinity for MHC class I results in a low effective concentration of H-2K^b^/S598 complex on the cell surface, the responding T cells should be high avidity, based, primarily, on *in vitro* studies [37]. While the relationship between level of surface peptide and avidity of the responding T cells generated in vivo is not as clearcut [38], there is no obvious explanation for how a peptide with low MHC class I binding also elicits a low avidity T cell response. This selection of only a subset of CD8 T cells capable of responding to S598 may partly explain why S598-primed cells do not recognize the Q600Y determinant.

The biological significance of the heteroclitic Q600Y determinant was shown by its ability to protect JHMV-infected mice from CTL escape at S510 and to diminish clinical disease. This was important to demonstrate because other studies, using tumor models, have shown that immunogenicity and tumor recognition are not necessarily concordant [39]. Mutations resulting in CTL escape occur most commonly in determinants that are exposed to high selective pressure [40]–[42] and outgrowth of CTL escape variants is efficiently suppressed by effective and rapid virus clearance [43]–[46], as occurs in mice infected with rJ.S_Q600Y_. Thus, even though CTL escape is not detected in normal mice infected with LCMV or influenza, escape does occur when mice transgenic for a single LCMV-specific TcR are infected with high amounts of virus [44],[47]. Under these conditions, the immune response is highly focused on a single CD8 T cell determinant and virus replication continues for extended periods of time, facilitating mutation at the targeted determinant.

In mice infected with wild type JHMV, the CTL response is functionally focused on S510 [21]; the Q600Y substitution effectively prevents CTL escape at either S510 or S598_Q600Y_ by the induction of a second high avidity CTL response. Mutations in S598_Q600Y_ were not detected even when the CTL response was directed primarily at this determinant (e.g. mice infected with rJ.S_W513R_+_Q600Y_). Consistent with this inability to readily tolerate mutations, we were unable to generate recombinant virus mutated in position Ile-4 (I601D,E,K,R,T) and recombinant virus mutated at Phe-5 (F602A) was highly attenuated (data not shown). The combination of induction of high avidity CTL and inability to tolerate mutation without adversely affecting virus fitness make S598_Q600Y_ an ideal target for the anti-JHMV CTL response. Further, the ala scanning results suggest that S598_Q600Y_-specific CTLs may more readily tolerate changes in the H-2K^b^/peptide complex, and this plasticity would also minimize the likelihood of CTL escape. In contrast, we have previously shown that introduction of the LCMV-specific GP33 determinant, which also elicits CTL with high functional avidity, into JHMV greatly diminished clinical disease but did not prevent CTL escape [22]. The GP33 determinant was introduced at a site in the genome that tolerated mutation and deletion and intact determinant was no longer present in virus by day 20 p.i. Collectively these results suggest that a response to a second determinant that elicits CTL exhibiting high functional avidity at early times p.i. results in enhanced suppression of virus replication, but its presence throughout the infection is required to protect against CTL escape.

In conclusion, we have demonstrated that crystal structures are useful in gaining an understanding of the basis of heteroclitic epitopes and can also prove valuable in guiding the rational design of “better” CTL epitopes. In our mouse system, immunization with the heteroclitic determinant resulted in the generation of unique populations of CTL that respond with high functional avidity to an otherwise modestly immunogenic viral epitope. The generation of unique populations of CTL that respond with high functional avidity to weakly immunogenic epitopes will be useful for the treatment and prevention of human infectious diseases. Our proposed structure-guided approach has direct application to HIV, HCV and other chronic infections in which virus persistence and CTL escape occurs. By modulating T cell immunity through prophylactic or therapeutic peptide-based vaccination, virus titers may be reduced and CTL escape and other consequences of viral persistence circumvented.

## Materials and Methods

### Mice

Specific pathogen-free B6 and BALB/c mice were obtained from National Cancer Institute (Bethesda, MD). To obtain infected mice in which CTL escape at S510 was detected, suckling B6 mice were infected intranasally with 2–4×10^4^ PFU of recombinant JHMV at 10 days of age and nursed by dams that were immunized with JHMV, as described previously [27]. For experiments comparing multiple JHMV variants, each litter served as an internal control: equal numbers of pups were infected with rJ and one to three recombinant variant viruses, depending on litter size. All animal studies were approved by the University of Iowa Animal Care and Use Committee.

### Intracellular cytokine staining

Mononuclear cells were harvested from the brains of acutely ill mice 7 days p.i. and analyzed for expression of IFN-γ by an intracellular cytokine staining protocol as previously described [28]. Unless otherwise noted, peptides corresponding to epitopes were used at a final concentration of 1 µM and cells were stimulated in the presence of 500 µM TCEP (Sigma, St. Louis, MO). Cells were analyzed using a FACScan flow cytometer (BD Biosciences, Mountain View, CA). Data sets were analyzed using FlowJo software (Tree Star, Inc, Ashland, OR). All antibodies and reagents were purchased from BD Pharmingen (San Diego, CA).

### Recombinant viruses

Recombinant wild-type and S510 and S598 variants of JHMV were generated as previously described [48],[49]. Briefly, overlapping extension polymerase chain reaction (PCR) was used to generate the S598_Q600Y_ and S510_W513R_ variants. Primers that overlapped the glutamine at residue 600 of the spike glycoprotein were (5′ to 3′) Q600Y fwd, ATGATCGCTGCTATATTTTTGCTAACATATTG; Q600Y rev, AATATGTTAGCAAAAATATAGCAGCGATCAT. Primers that overlapped the tryptophan at residue 513 were (5′ to 3′) W513R fwd, GTGAGTGTTCTCTTCGGAATGGGCCCCATTTGCGCTCGGC; W513R rev, AGCGCAAATGGGGCCCATTCCGAAGAGAACACTCAC. The outer primers for each targeted change were fwd, TGTTGATTGCGCCAGCAGCTACATTAG; and rev, ACCTACGGATTGAACGCTATCATTGAC. Underlined nucleotides correspond to the nucleotides encoding the Gln to Tyr and Trp to Arg substitutions within S598 and S510, respectively. Recombinant viruses encoding the variant epitope(s) were selected, propagated and titered as previously described [49]. At least two independent isolates of each recombinant virus were analyzed.

### One-step viral growth kinetics

Virus was inoculated onto confluent 17Cl-1 monolayers in a 12-well plate at a multiplicity of infection (MOI) of 1.0. Groups of cells were harvested at the indicated time points and total virus (cell-associated and cell-free) was titered as previously described [27].

### *In vivo* virus competition assay

Equal PFU (2–4×10^4^) of rJ and rJ.S_Q600Y_ were combined and inoculated intranasally into 5-week-old B6 and BALB/c mice. Total RNA was harvested from the brains of mice 7 days p.i. and the relative representation of WT (wild type) versus variant template was determined by RT-PCR and sequencing. This assay can specifically detect a given species of template when that species comprises at least 20% of a heterogeneous pool [50]. Primers used were (5′-3′): forward, AACCCCTCGTCTTGGAATAGGAGGTATGG; and reverse, CCTACGGATTGAACGCTATCATTGACTAAC. PCR products were sequenced directly by the University of Iowa DNA Core.

### Alanine scans and functional avidity determination

For alanine scanning, cells were stimulated *ex vivo* with the indicated concentration of native or variant peptide and stained for CD8 and IFN-γ as described above. Data were normalized to the frequency of cells that reacted to the unmodified S598 or S598_Q600Y_ peptides. For functional avidity determination, mononuclear cells were harvested from the brains of rJ or rJ.S598_Q600Y_-infected mice 7 days p.i. and stimulated *ex vivo* in the presence of EL-4 cells pulsed with tenfold dilutions of peptide corresponding to native S598 or S598_Q600Y_ epitopes. After 5.5 hours, cells were stained for intracellular IFN-γ as described above. For each epitope-specific population, data were normalized to the frequency of antigen-specific CTL detected using the highest titration of peptide (1 µM).

### Circular dichroism

CD spectra were measured on a Jasco 810 spectropolarimeter using a thermostatically controlled cuvette at temperatures between 30 and 90°C. Far-UV spectra were collected and analyzed as described [23].

### TcR Vβ chain usage

Cells were harvested from the CNS of mice 7 days p.i. and stimulated *ex vivo* with S598 or S598_Q600Y_ peptides. Cell aliquots were subsequently stained for CD8 (PE-Cy7-anti-CD8α) and Vβ (FITC-anti-Vβ2, 3, 4, 5.1/5.2, 6, 7, 8, 9, 10^b^, 11, 12, 13 or 14, BD-Pharmingen) followed by intracellular staining for IFN-γ (PE-anti-IFN-γ). Data were collected using a Becton Dickinson LSR II instrument at the University of Iowa Flow Cytometry Facility. Data are expressed as the proportion of antigen-specific CD8 T cells that express each Vβ chain.

### RNA sequence analysis

Total RNA was purified with TRIzol (Invitrogen, Carlsbad, CA) from the CNS of mice. The 1055 base pair region of the spike glycoprotein encompassing both S510 and S598 was amplified by RT-PCR and sequenced directly as previously described [19].

### Dendritic cell immunization and JHMV challenge

Bone marrow-derived DC were prepared, pulsed with peptides and injected into mice as previously described [51]. Briefly, 5×10^5^ LPS-matured DC were left uncoated, coated with S598 or S598_Q600Y_ peptides and injected via tail vein into groups of 4-week-old mice. Seven days following DC-vaccination, mice were infected intranasally with 4×10^4^ PFU of JHMV. Seven days following virus infection, brains were harvested from mice and the frequencies of epitope-specific CD8 T cells were determined by *ex vivo* stimulation and intracellular cytokine staining as described above. In independent studies, DC-S598 and DC-S598_Q600Y_ priming was verified by harvesting spleens from several mice seven days following DC-vaccination.

### Crystal structure of H-2K^b^/S598 and H-2K^b^/S598_Q600Y_ complexes

Crystals of H-2K^b^S598-Aba were grown at 21°C in 0.1 M cacodylate pH 6.6, 16% PEG (polyethylene glycol) 8,000, 0.2 M Ca(OAc)_2_, using a protein concentration of 9 mg/ml. Crystals were cryoprotected by stepwise equilibration against mother liquor containing 5, 10 and 15% glycerol and flash frozen by placing in a nitrogen stream. A 1.8 Å resolution dataset was collected on an in-house X-ray source. Crystals of H-2K^b^S598_Q600Y_-Aba were grown at 21°C in 0.1 M cacodylate pH 6.5, 13% PEG 8,000, 0.2 M Ca(OAc)_2_, using a protein concentration of 6 mg/ml. Crystals were cryoprotected by gradual equilibration against mother liquor containing 20% PEG 8,000 and 5% glycerol before flash freezing. A 2.8 Å resolution dataset was collected on an in-house source. The WT data were integrated in *MOSFLM* [52] and scaled/merged using *SCALA* [53]. The Q600Y variant data were processed using *HKL2000*. Both structures were solved by molecular replacement in *PHASER* [54], against previously solved H-2K^b^ complexes (PDBid's: 1G7Q and 1RJY, respectively). The resultant models underwent iterative cycles of refinement in *PHENIX* [55] and, *REFMAC5* [56] (restrained and TLS refinement) followed by model building/correction in *COOT* [57]. The solvent structures were built using *ARP/wARP* [58] and *COOT*. A summary of the processing and refinement statistics is presented in Table S1.

### Statistical analyses

Statistical significance was determined by nonpaired, two-sided Student's *t* test or chi-squared test, where indicated.

## Supporting Information

Figure S1 Immunogenicity of the Aba-modified, S598 peptidomimetic. (A) Intracellular IFN-γ staining of splenocytes from mice peripherally immunized with JHMV simulated ex vivo with peptides (1 µM) corresponding to the native or Aba-modified S598 determinant. (B) Native S598 peptide is more stimulatory when S598-specific CTL are stimulated in the presence of reducing agent. Splenocytes were harvested from JHMV-immune mice and reacted with native S598 peptide (1 µM) in the presence of 500 µM TCEP. Data in A represent the mean±SEM for 3 experiments. Data in B are representative of five independent experiments.
(0.25 MB TIF)

Figure S2 TCR Vβ chain usage and alanine scanning analysis. (A) Total mononuclear cells were harvested from mice infected with rJ or rJ.S_Q600Y_ and stimulated *ex vivo* with S598 and S598_Q600Y_ peptides, respectively. Following stimulation, aliquots of cells were surface stained for CD8 and the indicated Vβ chain followed by intracellular cytokine staining for IFN-γ. (B) CNS-derived cells from rJ.S_Q600Y_-infected mice were stimulated *ex vivo* with peptides corresponding to native S598 or S598_Q600Y_ epitopes. Following stimulation, cells were analyzed as described for (A). Data represent the fraction of IFN-γ+CD8+ T cells expressing each Vβ chain and are derived from cells pooled from 2–3 individual mice. (C) Alanine scanning of the native S598 determinant. CNS-derived mononuclear cells were recovered from rJ-infected mice 7 days p.i. and stimulated *ex vivo* in the presence of 500 µM TCEP and 1 µM of the indicated peptide then stained for CD8 and intracellular IFN-γ. Data are normalized to the frequency of epitope-specific cells detected when stimulated with the native S598 determinant. (D) Alanine scanning of the S598_Q600Y_ determinant. Cells were harvested and tested as described for B except in this case the cells originated from the rJ.S_Q600Y_-infected CNS and were stimulated with 10 nM S598_Q600Y_ peptide. (E) Alanine scanning of the S598 determinant recognized by S598_Q600Y_-primed, cross-reactive CTL. As in C, but cells were stimulated with 150 nM S598 peptide. For B–D, concentrations of peptide equivalent to 10× that required for half maximal stimulation were used; data are mean±SEM from four independent experiments. Note that the differential responses to RCAIFANI in panels C and D reflect the differing amounts of peptide used in the two assays.
(0.76 MB TIF)

Figure S3 Refined structures of WT and Q600Y S598-Aba bound to H-2K^b^. (A) View of the H-2K^b^ antigen binding cleft from above. The HC is shown as a cartoon representation and coloured slate. The peptide is in stick format with carbon atoms coloured yellow. The unbiased *F_o_-F_c_* map density for the peptide contoured at 2.5 σ is shown as a magenta mesh. (B) The same view as in A displaying key interactions (dashed lines) between H-2K^b^ and S598-Aba. Selected residues of the HC are drawn in stick format (slate carbon atoms) and ordered water molecules are shown as red spheres. Peptide residues are labelled in italics. C and D, Equivalencies to A and B, respectively, for the H-2K^b^/S598_Q600Y_-Aba structure. In these panels the HC is drawn in green and the peptide in cyan.
(4.07 MB EPS)

Table S1 Data collection and refinement statistics.
(0.06 MB DOC)

Table S2 Contacts between the S598 determinant and H-2K^b^.
(0.07 MB DOC)
