# Supplementary figures and images for: Prevention of Cytotoxic T Cell Escape Using a Heteroclitic Subdominant Viral T Cell Determinant
Source: PLoS Pathog. 2008 Oct 24;4(10):e1000186. doi: 10.1371/journal.ppat.1000186 (PMC2563037; doi:10.1371/journal.ppat.1000186)

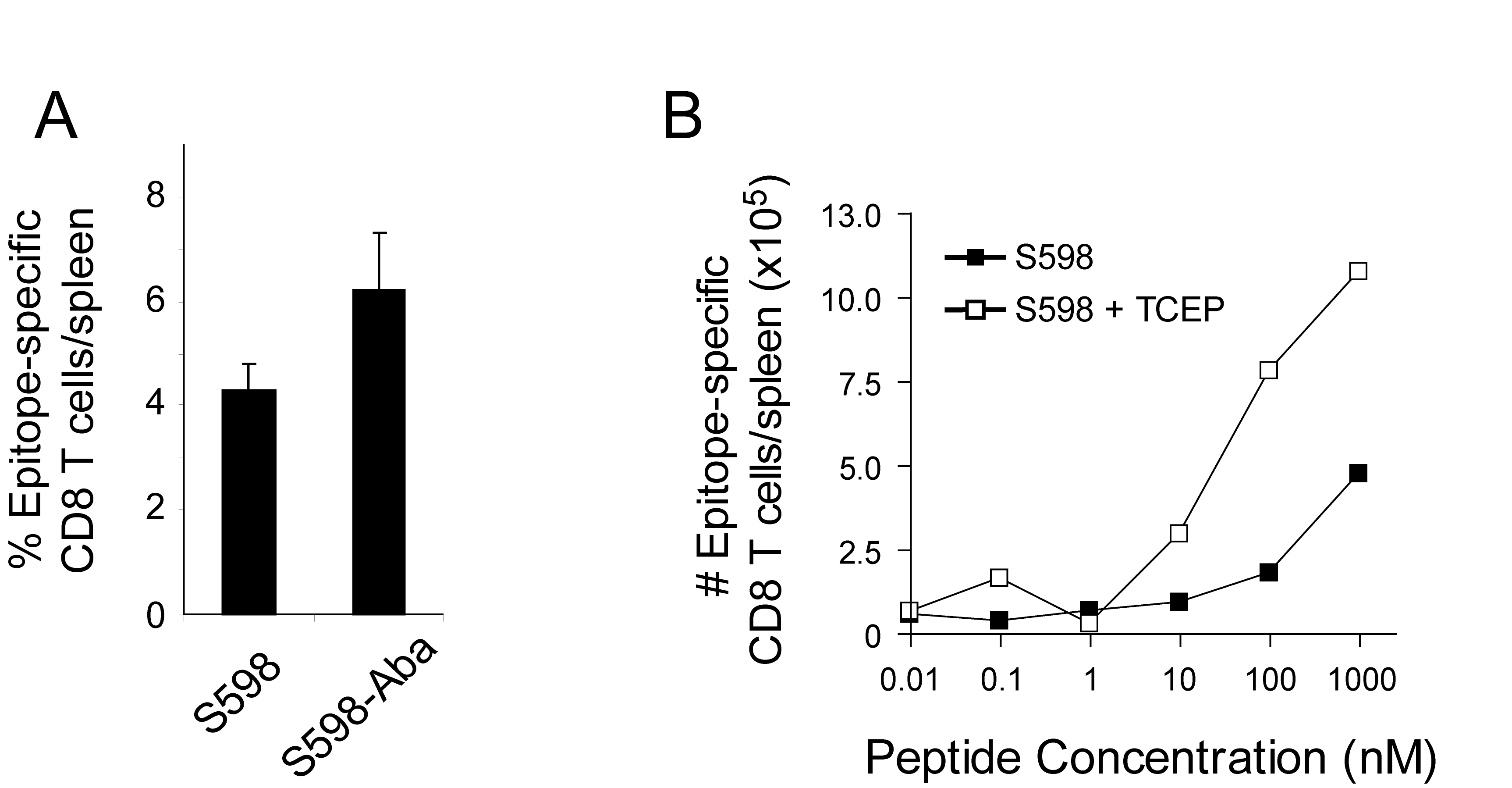

Supplement: Figure S1 — Immunogenicity of the Aba-modified, S598 peptidomimetic. (A) Intracellular IFN-γ staining of splenocytes from mice peripherally immunized with JHMV simulated ex vivo with peptides (1 µM) corresponding to the native or Aba-modified S598 determinant. (B) Native S598 peptide is more stimulatory when S598-specific CTL are stimulated in the presence of reducing agent. Splenocytes were harvested from JHMV-immune mice and reacted with native S598 peptide (1 µM) in the presence of 500 µM TCEP. Data in A represent the mean±SEM for 3 experiments. Data in B are representative of five independent experiments. (0.25 MB TIF) [file ppat.1000186.s001.tif]

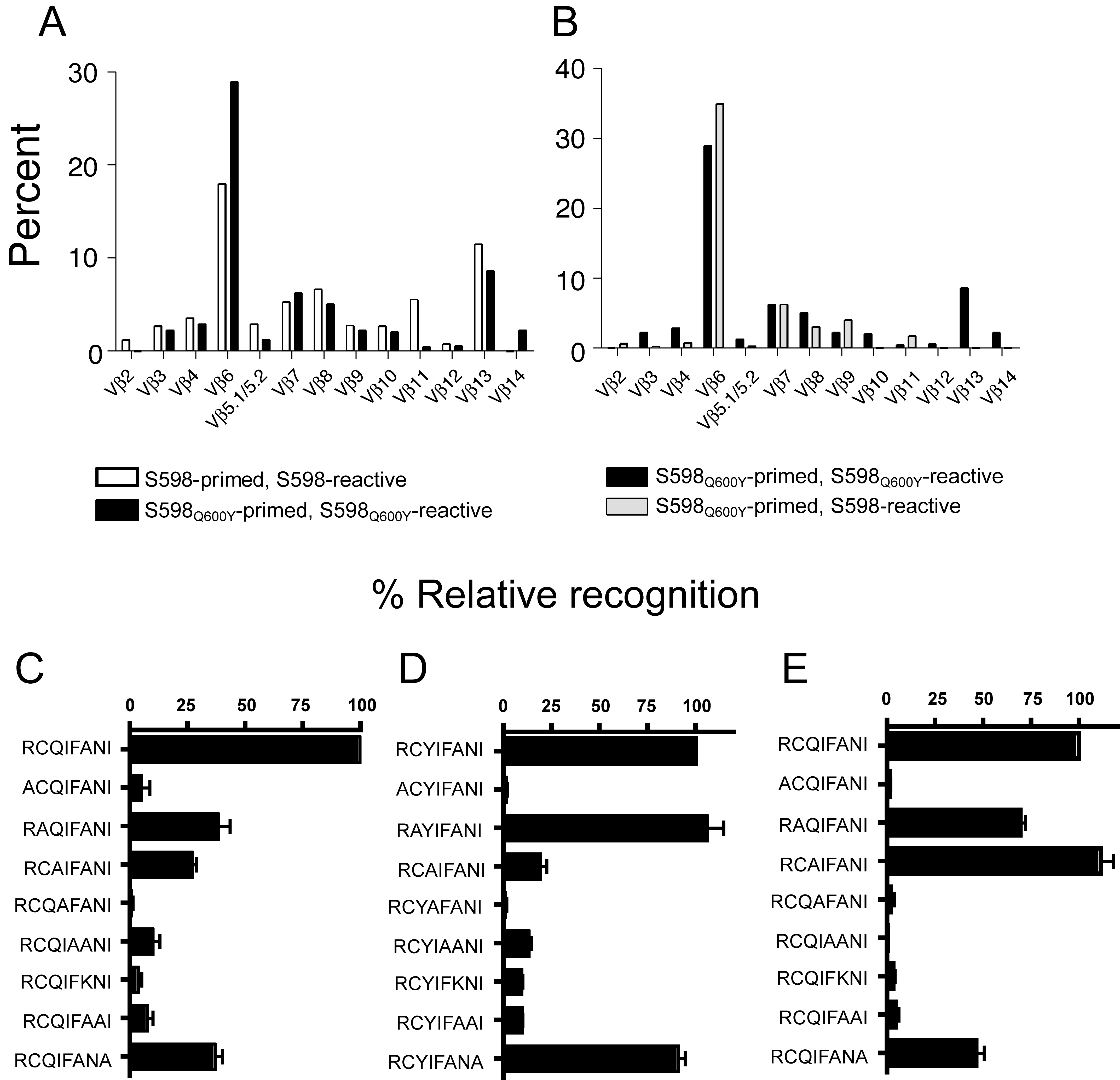

Supplement: Figure S2 — TCR Vβ chain usage and alanine scanning analysis. (A) Total mononuclear cells were harvested from mice infected with rJ or rJ.SQ600Y and stimulated ex vivo with S598 and S598Q600Y peptides, respectively. Following stimulation, aliquots of cells were surface stained for CD8 and the indicated Vβ chain followed by intracellular cytokine staining for IFN-γ. (B) CNS-derived cells from rJ.SQ600Y-infected mice were stimulated ex vivo with peptides corresponding to native S598 or S598Q600Y epitopes. Following stimulation, cells were analyzed as described for (A). Data represent the fraction of IFN-γ+CD8+ T cells expressing each Vβ chain and are derived from cells pooled from 2–3 individual mice. (C) Alanine scanning of the native S598 determinant. CNS-derived mononuclear cells were recovered from rJ-infected mice 7 days p.i. and stimulated ex vivo in the presence of 500 µM TCEP and 1 µM of the indicated peptide then stained for CD8 and intracellular IFN-γ. Data are normalized to the frequency of epitope-specific cells detected when stimulated with the native S598 determinant. (D) Alanine scanning of the S598Q600Y determinant. Cells were harvested and tested as described for B except in this case the cells originated from the rJ.SQ600Y-infected CNS and were stimulated with 10 nM S598Q600Y peptide. (E) Alanine scanning of the S598 determinant recognized by S598Q600Y-primed, cross-reactive CTL. As in C, but cells were stimulated with 150 nM S598 peptide. For B–D, concentrations of peptide equivalent to 10× that required for half maximal stimulation were used; data are mean±SEM from four independent experiments. Note that the differential responses to RCAIFANI in panels C and D reflect the differing amounts of peptide used in the two assays. (0.76 MB TIF) [file ppat.1000186.s002.tif]
