# Supplementary material for: Prevention of Cytotoxic T Cell Escape Using a Heteroclitic Subdominant Viral T Cell Determinant
Source: PLoS Pathog. 2008 Oct 24;4(10):e1000186. doi: 10.1371/journal.ppat.1000186 (PMC2563037; doi:10.1371/journal.ppat.1000186)
Supplement: Table S1 — Data collection and refinement statistics. (0.06 MB DOC) [file ppat.1000186.s004.doc]

Table S1. Data collection and refinement statistics

| Structure | | **H-2K^b^S598-Aba** | | **H-2K^b^S598-Q600Y-Aba** |
| --- | --- | --- | --- | --- |
| Wavelength (Å) | | 1.54178 | | 1.54178 |
| Resolution limits (Å)^a^ | | 24.46 – 1.80 (1.90 – 1.80) | | 27.00 – 2.80 (2.91 – 2.80) |
| Space group | | *P*2_1_ | | *P*1 |
| Unit-cell parameters (Å) | | *a* = 66.49, *b* = 89.54, *c* = 89.93 Å  β = 111.68° | | *a* = 66.71, *b* = 90.15, *c* = 91.97 Å  α = 81.11, β = 70.58, γ = 68.25° |
| No. observations | | 258,132 | | 109,505 |
| No. unique reflections | | 90,277 | | 48,224 |
| Mosaicity | | 0.80 | | 0.80 |
| Completeness (%)^a^ | | 99.6 (99.5) | | 95.8 (85.6) |
| *R*_merge_ (%) ^a,b^ | | 4.6 (38.5) | | 10.1 (30.3) |
| < *I*/σ(*I*) >^a^ | | 18.0 (2.0) | | 10.0 (2.1) |
| Multiplicity^a^ | | 2.9 (2.7) | | 2.3 (2.1) |
| R-factor (%)^c^ | 20.8 | | 20.7 | |
| R-free (%)^d^ | 25.1 | | 25.3 | |
| Number of atoms:  - protein  - peptide  - water  - other | 6,081  128  607  60 | | 11,994  268  136  - | |
| Average B-factor (Å^2^):  - protein  - peptide  - water  - other | 23.7  19.4  30.5  44.9 | | 47.6  47.5  32.7  - | |
| rmsd bonds (Å) | 0.017 | | 0.019 | |
| rmsd angles (°) | 1.61 | | 1.66 | |

^a^ Values in parentheses refer to the highest resolution bin.

^b^ R_merge_ = s_hkl_ Σ_i_ | I_hkl, i_ - <I_hkl_> | / Σ_hkl_ <I_hkl_>

^c^ R_factor_ = ( Σ | |F_o_| - |F_c_| | ) / ( Σ|F_o_| ) - for all data except as indicated in footnote d.

^d^ 5% of data was used for the R_free_ calculation (see footnote c).
