# Supplementary material for: Prevention of Cytotoxic T Cell Escape Using a Heteroclitic Subdominant Viral T Cell Determinant
Source: PLoS Pathog. 2008 Oct 24;4(10):e1000186. doi: 10.1371/journal.ppat.1000186 (PMC2563037; doi:10.1371/journal.ppat.1000186)
Supplement: Table S2 — Contacts between the S598 determinant and H-2Kb. (0.07 MB DOC) [file ppat.1000186.s005.doc]

Table S2. Contacts between the S598 determinant and H-2Kb

| **Peptide Residue** | | **Type of Contact** | | **Contact Residues^a,b^** | | |
| --- | --- | --- | --- | --- | --- | --- |
|  |  |  |  | **H-2K^b^S598-Aba** | | **H-2K^b^S598_Q600Y_-Aba** |
| Arg-1 | | Van der Waals^c^ | | L5, Y7, E63, K66, Y159, T163, W167, Y171, *Q3* | | Y7, E63, K66, Y159, T163, W167, Y171, *Y3* |
| Arg-1^N, O^ | | Hydrogen bond^d^ | | Y7^Oη^, Y159^Oη^, Y171^Oη^ | | Y7^Oη^, Y159^Oη^, Y171^Oη^ |
| Arg-1^Nε^ | | Hydrogen bond | | E63^Oε2^ | | E63^Oε2^ |
| Aba-2 | | Van der Waals | | Y7, E24, E63, K66, Y159, *I4*, *F5* | | Y7, E24, E63, K66, Y159, *I4*, *F5* |
| Aba-2^N,O^ | | Hydrogen bond | | K66^Nζ^ | | K66^Nζ^ |
| Aba-2^N^ | Water-mediated^e^ | | Y45^Oη^, E63^Oε1^ | | - | |
| Gln-3/Tyr-3 | Van der Waals | | N70, S99, Q114, L156, Y159, , *R1*, *F5* | | N70, S99, Q114, E152, R155, L156, Y159, *F5* | |
| Gln-3^N,O^/Tyr-3^N,O^ | Hydrogen bond | | N70^Nδ2^ | | N70^Nδ2^ | |
| Gln-3^Oε1,Nε2^/Tyr-3^Oη^ | Hydrogen bond | | S99^Oγ^, Q114^Oε1^ | | E152^Oε1,Oε2^ | |
| Gln-3^N^/Tyr-3 | Water-mediated | | S99^Oγ^, Y159^Oη^ | | - | |
| Ile-4 | Van der Waals | | K66, N70, R155, *Aba2*, *A6* | | K66, N70, R155, *Aba2*, *A6* | |
| Ile-4^O^ | Hydrogen bond | | R155^Nη1,Nη2^ | | R155^Nη1,Nη2^ | |
| Phe-5 | Van der Waals | | V9, N70, F74, V97, Y116, R155, *Aba2*, *Q3* | | N70, S73, F74, V97, S99, Q114, Y116, R155, *Aba2*, *Y3*, *N7* | |
| Phe-5^N^ | Hydrogen bond | | N70^Oδ1^ | | N70^Oδ1^ | |
| Phe-5^O^ | Water-mediated | | N70^Oδ1^, S73^Oγ^ | | - | |
| Ala-6 | Van der Waals | | Y116, W147, E152, R155, *I4*, *I8* | | D77, Y116, W147, E152, R155, *I4*, *I8* | |
| Ala-6^N,O^ | Water-mediated | | D77^Oδ2^, Q114^Oε1^, Y116^Oη^ | | - | |
| Asn-7 | Van der Waals | | S73, V76, D77, K146, W147 | | S73, D77, W147, *F5* | |
| Asn-7^O^ | Hydrogen bond | | W147^Nε1^ | | W147^Nε1^ | |
| Asn-7 ^Nδ2^ | Hydrogen bond | | S73^O^, D77^Oδ1^ | | S73^O,Oγ^, D77^Oδ1^ | |
| Asn-7 ^Oδ1^ | Water-mediated | | S73^Oγ^ | | - | |
| Ile-8 | Van der Waals | | D77, T80, L81, Y84, Y116, T143, K146, W147, *A6* | | D77, L81, Y84, Y116, Y123, T143, W147, *A6* | |
| Ile-8^N,O,OXT^ | Hydrogen bond | | D77^Oδ1^, Y84^Oη^, T143^Oγ1^, K146^Nζ^ | | D77^Oδ1Oδ2^, Y84^Oη^, T143^Oγ1^ | |
| Ile-8^O^ | Water-mediated | | D77^Oδ1^, T80^Oγ1^ | | - | |

^a^ Atomic contacts determined using the CCP4i implementation of *CONTACT* (1) and a cutoff of 4 Å. All contacts listed were observed in at least half the copies in the asymmetric unit.

^b^ Contacts between peptide residues are labeled in italics.

^c^ Van der Waals interactions defined as non-hydrogen bond contact distances of 4 Å or less.

^d^ Hydrogen bond interactions are defined as contact distances of 3.5 Å or less, between suitable atoms at appropriate angles (2), as determined in Coot.

^e^ Water mediated hydrogen bond (see footnote d).

* 1) Collaborative Computational Project, Number 4. 1994. "The CCP4 Suite: Programs for Protein Crystallography". Acta Cryst. D50, 760-763

2) Baker, EN & Hubbard, RE (1984). Hydrogen bonding in globular proteins. Prog Biophys Mol Biol, 44, 97-179.
